# Current Advances and Future Perspectives on Mesenchymal Stem Cell-Derived Extracellular Vesicles in Alzheimer's Disease

**DOI:** 10.14336/AD.2023.1206

**Published:** 2024-10-01

**Authors:** Wenjing Zhang, Russell Uyemura, Kun Zhong, Rui Guo, Li Zhong

**Affiliations:** ^1^College of Life Sciences, Institute of Life Science and Green Development, Hebei University, Baoding 071002, China.; ^2^College of Osteopathic Medicine of the Pacific, Western University of Health Sciences, Pomona, California 91766, USA.; ^3^American Center of Stem Cell Research and Regenerative Medicine, Farmington Hills, Michigan 48336, USA

**Keywords:** mesenchymal stem cells, extracellular vesicles, Alzheimer's disease

## Abstract

The incidence of Alzheimer's disease (AD) has been increasing in recent years as the world's population ages, which poses a significant challenge to public health. Due to the complexity of pathogenesis of AD, currently there is no effective treatment for it. In recent years, cell and gene therapy has attracted widespread attention in the treatment of neurodegenerative diseases. Mesenchymal stem cell-derived extracellular vesicles (MSC-EVs) represent a novel cell-free therapy with numerous advantages over cell-based therapies owing to their low immunogenicity and high safety profile. We summarize recent progress in the application of EVs for treating AD and the specific mechanisms and outline the underlying mechanisms. We also explore various methods for optimizing the function of MSC-EVs, including gene editing, modifying stem cell culture conditions and peptide modification. In addition, we discuss the therapeutic potentials of MSC-EVs, as well as the obstacles that currently impede their clinical utilization.

## 1. Introduction

Alzheimer's disease (AD) is the leading cause of dementia, which is a neurodegenerative disease correlated with neuronal impairment [1]. In the United States, AD was ranked as the sixth leading cause of death in 2019 and has been recently observed to have a higher mortality rate with COVID-19 coinfection [2]. By the year of 2060, it is estimated that approximately 13.8 million people in the United States will be affected AD, resulting in substantial healthcare expenses for both families and society [3]. Pathologically, AD manifests as abnormal accumulation of amyloid β (Aβ) plaques and neurogenic fiber tangles (NFTs) that are composed of phosphorylated tau proteins [4]. Soluble amyloid β oligomers (AβO) causes neuronal dysfunctions that activate neuroglia cells in the brain, triggering chronic neuroinflammation [5]. Microglia serve as the primary immune cells of the innate immunity system within the brain [6]. These cells play an important role in AD by participating in the phagocytosis of Aβ plaques and are involved in the inflammatory response [7, 8].

Among the drugs approved by the US Food and Drug Administration (FDA), the cholinesterase inhibitors (donepezil, galantamine, rivastigmine) and N-methyl-d-aspartate receptor antagonist (memantine) are able to palliate cognitive disorder in patients by inhibiting the decompression of acetylcholinesterase [9]. However, there are no effective treatments and medicine in the world to cure AD fundamentally, rather than temporarily relieving symptoms in the short-term. Therefore, new approaches are urgently needed for the treatment of AD [10]. In recent years, Mesenchymal stem cells (MSCs) have been considered as a new therapy for AD because of their low-immune rejection, anti-inflammatory effect, and extensive differentiation properties for clinical trials [11-14]. In neurodegenerative disorders, MSCs primarily function through three mechanisms: secretion of growth factors for immunomodulation, inhibition of neuroinflammation, and paracrine release of extracellular vesicles (EVs) [15]. Wei *et al*. have observed that MSCs inhibit AD mainly through paracrine EVs, rather than the cells [16]. EVs contain proteins, lipids, DNA, mRNA and miRNA, which play a pivotal role in cellular communication by transporting these active substances and regulating various metabolic pathways, including cell proliferation, apoptosis, autophagy and other processes [17-23]. We summarize how MSC-EVs can be utilized as a novel treatment for AD.

## 2. Mesenchymal stem cell-derived extracellular vesicles (MSC-EVs)

MSCs are pluripotent stem cells with multidirectional differentiation and possess self-renewal capabilities that enable to migrate towards damaged tissue repair sites [24]. Because of their ability to differentiate into neuronal cells, MSCs have received wide attention for their therapeutic potential in neurological diseases [25, 26]. Although research on stem cell therapy has continued for the past two decades, safety risks, regulatory barriers and ethical controversies still pose significant challenges for its development and clinical application [27]. Numbers of studies have recently shown that MSCs exerts its therapeutic effects mainly through paracrine mechanisms and the secretion of EVs [28, 29].

EVs are double-membrane vesicles released by cells and can be classified based on their biological origin into apoptotic bodies, exosomes, and ectosomes. Apoptotic bodies are the largest, ranging in size from 50 nm to 5000 nm [30]. Exosomes are ranging in size from 30 nm to 150 nm and have a structure that resembles a pancake with a concave center when observed under transmission electron microscope (TEM) [31, 32]. During the transformation of endosomes into mature multivesicular endosomes (MVE), the endosomal membrane buds and the MVE fuses with the cell membrane to release ILV outside the cell, which in turn forms exosomes [33, 34]. Ectosomes are formed by outward budding of the membrane and range in size from 100 nm to 1000 nm [35].

The application of stem cells has many limitations such as undirected differentiation and malignant metastasis. There is the possibility of immune rejection of stem cell transplantation, due to the expression of major histocompatibility complex (MHC) I that exacerbates the persistence of inflammation [36, 37]. Compared to stem cell transplantation, MSC-EVs have the advantage of being safer, more convenient, more effective, and less ethical issues. Tang *et al*. demonstrated that MSC-EVs were more effective than MSCs in promoting chondrocyte regeneration and bone damage repair for osteoarthritis. The reason is probably that EVs can be taken up by chondrocytes and directly affect the signaling pathways of target cells [38]. In AD-related studies, it was shown that MSC-EVs were able to reduce Aβ stimulation of microglia and attenuate the inflammatory response more effectively than MSCs [39]. MSC-EVs can interact with target cells by fusing with the plasma membrane and delivering the bioactive molecules directly, such as DNA, mRNA, miRNA, cytokines and enzymes [40, 41]. Currently, MSC-EVs have been widely studied for Alzheimer's disease, Parkinson's disease, multiple sclerosis, Huntington's disease, and several other neurodegenerative diseases [42-45].

## 3. Mechanism of action of MSC-EVs in AD

For the pathogenic mechanism of AD, there are several hypotheses: Aβ hypothesis, tau hypothesis [46], cholinergic hypothesis (CH) [47], neuroinflammatory hypothesis [48], and calcium hypothesis [49]. Microglia, as resident macrophages of the central nervous system, its abnormal proliferation and activation often occur around amyloid plaques in the brain [50].

**Figure 1.**
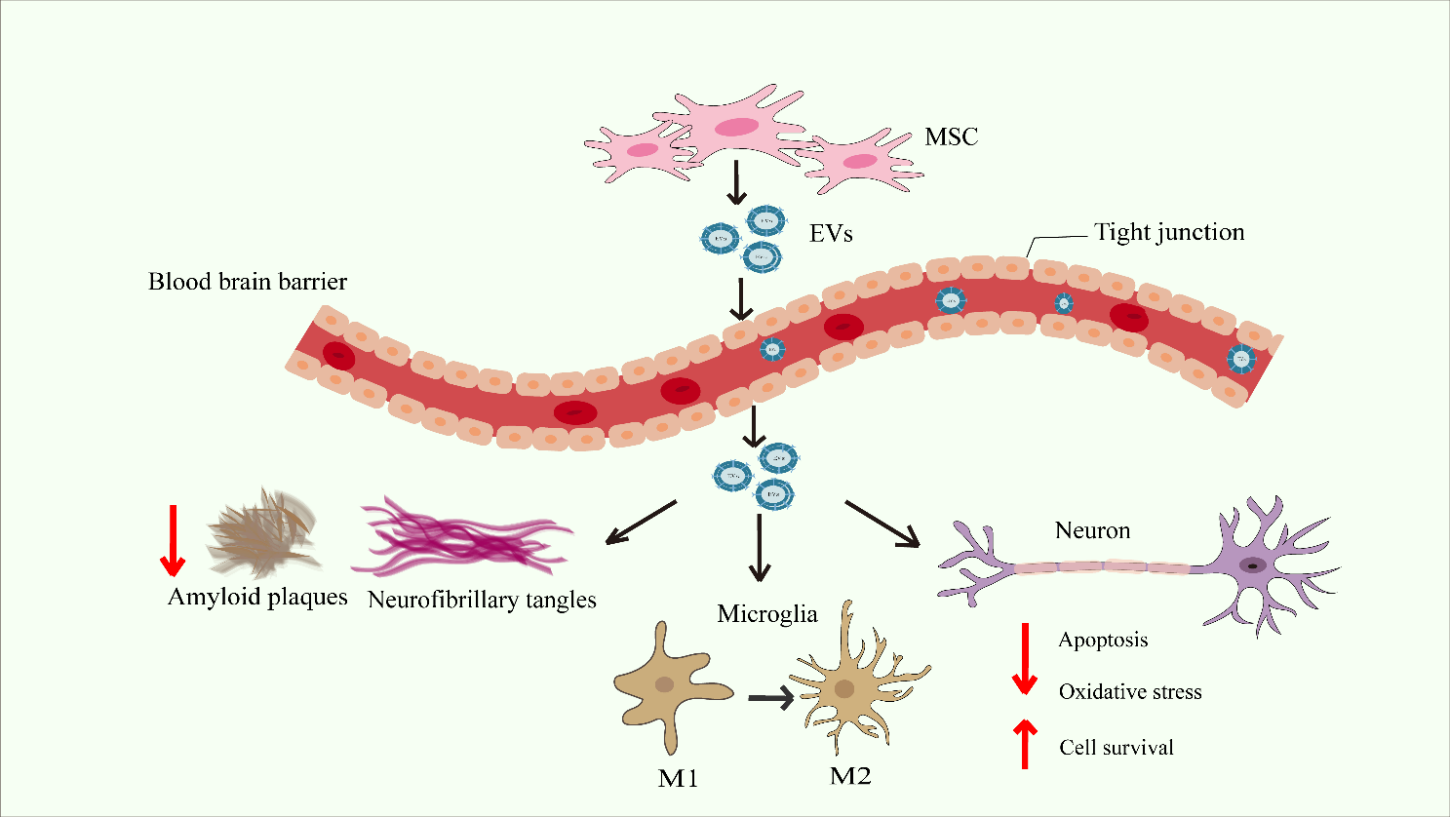
Mechanism of action of MSC-EVs in AD. MSC-EVs can cross the blood-brain barrier, and reduces the accumulation of Aβ and p-tau in the brain, promoting the transformation of microglia to the M2, and has beneficial effects on neurons. MSC, mesenchymal stem cell; EVs, extracellular vesicles; M1, pro-inflammatory phenotype of microglia; M2, anti-inflammatory phenotype of microglia.

Biochemical and behavioral tests are employed in AD mouse models to assess the progression of the disease. Typical pathology is characterized by the presence of Aβ plaques, NFTs, and neuronal death caused by inflammation in brain tissue [51]. The majority of studies have primarily concentrated on investigating cognitive and motor impairments in AD mice. Moreover, there is growing evidence suggesting that social withdrawal and depressive behaviors increase with the development of pathology [52]. For the past few years, AD mouse model has been utilized by multiple basic and preclinical studies. Common mouse models include 5×FAD, APP/PS1, 3×Tg-AD, SAMP8, and J20 mice, as well as normal mice treated with drugs to trigger disease symptoms [53-58]. Various studies have observed that MSC-EVs can cross the blood-brain barrier (BBB) to improve cognitive impairment in AD in several ways, including alleviating pathological features, decreasing inflammation, and immunomodulation [59-61] (Fig. 1). The recent research progress on MSC-EVs for AD is summarized in Table 1.

### 3.1EVs can cross the BBB

BBB is composed of endothelial cells, pericytes, and astrocytes that form the capillaries [62, 63]. BBB integrity is strictly controlled under physiological conditions by the cells within the neurovascular unit and the basement membrane. In addition, the tight junctions (TJs) between the brain endothelium and other cells limit the osmotic exchange of macromolecules and polar molecules between the plasma and brain parenchyma to maintain central nervous system (CNS) homeostasis (Fig. 1) [62, 64]. The delivery of AD drugs to the brain is limited by BBB, as pharmaceuticals with molecular weights greater than 400 Da are largely impermeable [65]. EVs, as nanoscale vesicles for intercellular communication, can cross the BBB and accumulate within the brain [60, 61, 65] (Fig. 1). While the mechanism in which EVs cross the BBB is not fully understood, one possible pathway is active transport, from one side of the cell to the other using biomolecule carriers on the cell membrane [66, 67].

### 3.2 Pathological features of MSC-EVs relieving AD

In the Aβ hypothesis, mutant presenilin (PSEN) and amyloid beta precursor protein (APP) leads to the accumulation of Aβ42 that forms cortical plaques in the brain [68]. After Aβ deposition occurs, it promotes extensive tau protein diffusion from the medial temporal lobe to neocortical areas [69]. Tau accumulation within neurons leads to NFTs that causes neuronal death. This triggers neural deformation and causes the organism to develop cognitive impairment [70]. Studies have shown that MSC-EVs can be taken up directly by microglia which further inhibits Aβ accumulation [12, 71, 72], reduces Aβ plaques and downregulates hyper-phosphorylated tau in hippocampus of 5×FAD after intranasal administration, and subsequently rescues synaptic deficits [42, 73]. Neprilysin (NEP), a vital enzyme in Aβ degradation, was found to be significantly reduced in AD patients. Recent studies have shown that ADSCs-EVs carry active NEP that can be transferred to microglia to promote Aβ degradation, and further mitigate neurological damage and memory deficits of APP/PS1 [74-76]. Lim *et al*. demonstrated that hUCMSCs-EVs containing cytokines such as galactose lectin-3 (GAL-3) and hepatocyte growth factor (HGF) have the ability to enhance the recovery of damaged neuronal cells by reducing phosphorylated tau and promoting synaptic plasticity [77, 78]. In summary, MSC-EVs have the potential therapeutic value to alleviate the pathological symptoms, memory and motor deficits of AD (Fig. 1).

### 3.3 MSC-EVs are involved in immune and inflammatory regulation

Aggregation of misfolded proteins in the brain triggers an innate immune response that releases inflammatory mediators and accelerates the pathogenesis of AD [79]. Microglia plays a major role in immune process as single-cell transcriptome analysis and genome-wide association analysis (GWAS) revealed that AD-related risk genes, such as apolipoprotein E (ApoE) and triggering receptor expressed on myeloid cells 2 (TREM2) [80-83]. Microglia are heterogeneous cells that are typically divided into two types. M1 is the pro-inflammatory phenotype, that is cytotoxic, while M2 is the anti-inflammatory phenotype that is associated with reparative functions (Fig. 1) [84-86]. Perets *et al*. suggested that when AD mice were intranasally treated with EVs, the EVs accumulated to inflamed areas of brain pathologies and migrated to highly inflamed areas, positively correlating with inflammatory signaling. The results of brain slices showed that EVs enter the brain and mainly co-localize with neurons uptake [87]. EVs can improve the inflammatory response in the brain, inhibit neuronal apoptosis and promote neuroregeneration to improve the progression of AD [88, 89]. MSC-EVs could regulate microglia polarization by increasing the ratio of the M2 anti-apoptotic phenotype and attenuates neuro-inflammation [88, 89]. hUCMSCs-EVs could inhibit the release of pro-inflammatory factors and transfer miRNAs to regulate cellular metabolic processes, such as oxidative stress, mitochondrial autophagy, and apoptosis [90, 91]. In addition, co-culture of human amniotic fluid stem cell EVs (hAFSC-EVs)-treated microglia with SH-SY5Y cells significantly inhibited oxidative stress and apoptosis in neuronal cells [92]. hUCMSCs-EVs enhanced forkhead box transcription factor 3a (FOXO3a) expression that improved mitochondrial autophagy and protected microglia from pyroptosis [93]. Furthermore, ADSC-EVs modulated TREM2 by delivering circ-Epc1 frontal expression and altered microglia M1/M2 polarization to improve cognitive performance in a mouse model of AD.

**Table 1:** Recent research progresses on MSC-EVs in AD.

Source	Animal models	Method of administration	EVs dose	Treatment time	Results and mechanism of action	Ref.
BMSCs	APP/PS1	Intravenous	6 mg/kg	Every other day for 2 weeks (30w)	Decreases Aβ plaques in cerebral cortex and hippocampus; enhances cognitive abilities; inhibits neuronal cell apoptosis; promotes neuroprotection and regeneration.	Zhu Y, *et al*., 2023 [144].
BMSCs	C57BL/6 mice by lateral ventricular injection Aβ (1-42)	Intravenous	100 µg/mice	every two days for 2 weeks.	Enhances mitochondrial autophagy; reduces inflammatory response; improves cognitive abilities.	Xu F, *et al.*, 2022 [110].
BMSCs	C57BL/6 mice by intracerebroventricular injection of streptozotocin (STZ)	Lateral ventricle administration and intravenous	0.5 μg (lateral ventricle) 250 μg (intravenous)	5 consecutive days (18w).	Inhibits the neuroglial cell activation; increases preference index of novel objects; reduces Aβ accumulation and Tau hyperphosphorylation in the hippocampus.	Liu S, *et al*. 2022 [145].
BMSCs	APP/PS1	Intracerebroventricular injection	1×10^5^ particles	every two weeks, twice (13m).	EVs transfer miR-146 into astrocytes, inhibits NF-kB signaling pathway and ameliorates astrocytic inflammation.	Nakano M, *et al*., 2020 [146].
BMSCs	3xTg	Intranasal	30 μg	21 consecutive days (7m).	Promotes the transformation of the M2 anti-inflammatory phenotype in microglia; increases the density of dendritic spines.	Losurdo M, *et al*., 2020 [147].
ADSCs	APP/PS1	Intranasal	1 mg/kg	every two days for 2 weeks (9m).	Relieves neuronal damage; promotes neuronal regeneration synaptic growth; improves memory impairment.	Ma X, *et al*., 2020 [76]

MSC-EVs, mesenchymal stem cell-derived extracellular vesicles; AD, Alzheimer's disease; BMSCs, bone marrow mesenchymal stem cells; ADSCs, adipose mesenchymal stem cells.

### 3.4 miRNAs in MSC-EVs are involved in signaling regulation

miRNAs are a class of small noncoding RNAs that regulate cellular metabolic processes through post-transcriptional modifications of genes [94]. They can be transferred between cells via EVs, participate in cellular communication, and regulate a variety of metabolic processes [95, 96]. In a recent study, the level of miR-146a-5p was lower in the cerebrospinal fluid of AD patients in comparison to healthy donors. Hua *et al*. have found that hUCMSCs-EVs containing miR-146a-5p improve neuroinflammation by regulating the downstream target gene TNF receptor associated factor 6 (TRAF6) to promote autophagy of microglia and inhibit pyroptosis [90, 97]. PTEN is a regulator of the PI3K signaling pathway that controls neuronal cell growth, division and differentiation [98, 99]. It was observed that miR-99b-3p contained in EVs can modulate microglia activation, autophagy and attenuate inflammatory responses by inhibiting PI3K/AKT/mTOR activation [100]. Ferroptosis, as a new mode of cell death has been closely associated with brain diseases [101]. Wang *et al*. found that miR-760-3p was abundant in ADSC-EVs and could reduce neuronal death by suppressing the expression of glutathione-specific gamma-glutamyl-cyclotransferase1 (CHAC1), a key gene for ferroptosis [102]. miR-124-3p in BMSC-EVs was found to exert neuroprotective effects by inhibiting the p38 MAPK signaling pathway and reducing glutamate-mediated excitotoxicity [103]. In general, the above studies indicate that miRNAs contained in MSC-EVs can participate in neuroprotection by regulating intercellular communication.

## 4. Optimizing the function of EVs

**Figure 2.**
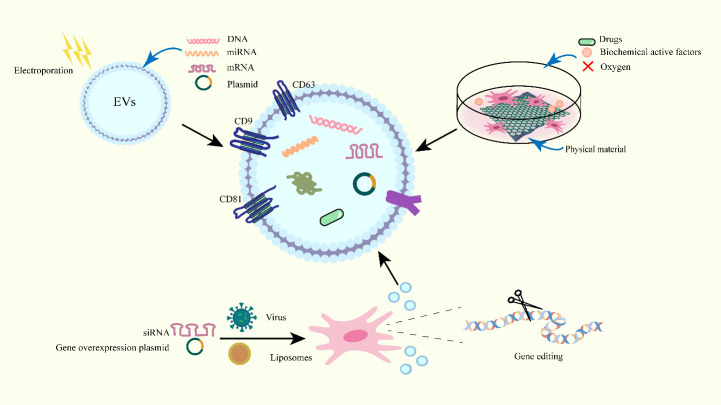
Optimizing the function of EVs. Remodeling EVs is achieved by importing active substances by electroporation, including proteins, lipids, DNA, mRNA and miRNA. Altering the culture conditions of parent cells to secrete functionalized EVs, EVs can be used as carriers to transport drug molecules. Use viruses or liposomes as vectors for constructing stably transfected stem cells, resulting in endogenous modifications of secrected EVs.

Large quantities of EVs are required for preclinical studies, but the current yields of EVs obtained by conventional methods are far from adequate [104, 105]. On the other hand, to achieve more efficient and precise drug delivery using EVs, the targeting of EVs *in vivo* should be improved [106]. As a result, the clinical translation of EVs receives multiple constraints. Currently, EVs as an adjuvant modified or combined with other technologies to overcome its limitations in clinical translation (Fig. 2) [107].

### 4.1 Engineered EVs

Engineered EVs refer to the use of genetic engineering techniques to edit various genes to enhance their specific function. Researchers typically use viral vectors for gene editing and constructing stably transfected stem cells, resulting in endogenous modifications of secreted EVs (Fig. 2) [108]. Engineered EVs can improve the therapeutic benefits of MSC-EVs by enhancing targeting, anti-inflammatory effects, increasing stem cells proliferation and differentiation.

Recently, researchers utilized CNS-specific rabies virus glycoprotein (RVG) to enhance the targeting of MSC-EVs to the cortex and hippocampus. It was observed that the expression levels of Aβ was significantly reduced in the brains of APP/PSEN mice that were intravenously injected with MSC-RVG-EVs. Furthermore, astrocyte activation was significantly reduced compared with normal MSC-EVs [109]. The overexpression of tyrosine phosphatase-2 (SHP2) in MSC-EVs were able to cross the BBB more efficiently, promoting SHP2 delivery to the brain, and inducing mitochondrial autophagy in neuronal cells. These EVs also ameliorate synaptic loss and cognitive decline in mice, with mitigating NLRP3 inflammatory vesicle activation, neuronal cell apoptosis and neuroinflammation [110]. Overexpression of the zinc finger E-box binding homeobox 2 protein (Zeb2) and the scaffolding protein Axin2 in EVs was observed to promote synaptic remodeling by regulating the target protein SRY-related HMG-box 10 protein (SOX10), modulating the Wnt/β signaling pathway, and promoting proliferation of neural stem cells [111].

In addition, EVs can be equipped with siRNA delivery for gene therapy *in vivo* (Fig. 2). Evers and his team merged EVs and liposomes to form EV-liposome hybrid nanoparticles, which can carry siRNA and deliver it to different cell types. The hybrid particles retain the characteristics of high RNA loading capacity and delivery efficiency [112]. ADSC-EVs containing siRNA against NF-κB pathway can accelerates skin repair in skin damage and reduces the expression of inflammatory factors [113]. Kang *et al*. coupled EVs to cardiac-targeting peptide (CTP) to form CEVs, which is loaded with siRNA that targets NADPH oxidase 4 (NOX4), a key gene that causes cardiac hypertrophy. Targeting CEVs-siNOX4 to the heart improved cardiac function, reduced fibrosis and cardiac hypertrophy [114]. Therefore, engineered EVs have great potential for the development in disease therapy.

### 4.2 EVs act as drug carriers

Due to their nanoscale structure and biocompatible characteristics, MSC-EVs can effectively load drugs and promote efficient uptake by target cells (Fig. 2). For example, lignocaine (LUT) is a plant flavonoid with anti-inflammatory effects and paclitaxel (PAC) is a widely used anti-cancer drug. However, both of them are limited by their poor water solubility, decreasing the efficiency of being absorbed by cells. When combined with MSC-EVs, LUT and PAC were found to increase their solubility and had improved therapeutic effects. Loading LUT with vesicles revealed a significantly higher uptake rate of LUT-EVs, which exhibited enhanced anti-fibrotic effects in *in vivo* experiments [115]. Transferring PAC into MSC-EVs by electroporation and incubating them with Hela cells demonstrated that EVs-PAC induced increased apoptosis in cervical cancer cells and inhibited the epithelial-mesenchymal transition (EMT) process [116, 117]. Similarly, hydrogen sulfide (H2S), an anti-inflammatory factor, combined with MSC-EVs, was observed to inhibit apoptosis and mitigate neurological damage caused by oxidative stress through downregulating TNF-α, miR-155 and Akt phosphorylation levels [118].

### 4.3 Altering the culture conditions of MSCs

In current studies, the low yield and poor activity of EVs are the major limiting factors for their widespread use in clinical medicine. Many studies have shown that changing the culture conditions of MSCs and pretreating these cells can influence the production and activity of EVs, regulating metabolic processes more effectively (Fig. 2).

#### 4.3.1 Hypoxic pretreatment

Different from the internal environment, MSCs are in a hypoxic environment *in vivo*. Numerous studies have demonstrated that hypoxic treatment promote changes in their expression profile and biological functions of EVs (Fig. 2) [119]. MSC-EVs obtained after hypoxic pretreatment (HEVs) were observed to improve learning and memory in APP/PS1. Furthermore, sequencing of EVs revealed alterations in noncoding RNAs expressions, such as miRNA and circRNA. The potential mechanism may lie in the restoration of synaptic dysfunction and modulation of inflammatory responses through regulation of miR-21, miR-216a-5p to alleviate ER stress and inhibition of p38 MAPK signaling [14, 53, 107, 120]. For example, Circ-Epc1 was being upregulated in HEVs and modulated microglia M1/M2 polarization by acting on TREM2 to improve cognitive impairment in AD mice [14].

#### 4.3.2 Pre-treatment of biochemical components

Inflammatory factors such as interferon-γ (IFN-γ) and tumor necrosis factor-α (TNF-α) are able to upregulate the expression of MHC II in MSCs and enhance immunosuppression [121]. After pretreatment with IFNγ, high-throughput sequencing results showed that the expressions of miR-27b-3p and miR-149-5p were significantly decreased and the anti-inflammatory ability was significantly increased in ADSC-EVs [122]. Melatonin is an endogenous hormone involved in apoptosis, autophagy, and inflammatory responses [123, 124]. Recent studies found that anti-inflammatory-related factors such as let-7b-5p, miR-23a-3p, and miR-100-5p were significantly upregulated in EVs secreted after melatonin pretreatment of MSCs, which resulted in the alleviation of the Wnt, AMPK, and Ras signaling pathways by targeting chronic inflammatory response [123, 125]. Ye *et al*. found that MSC-EVs released after infarct preconditioning could reduce apoptosis and promote cerebrovascular remodeling by altering miRNA expression and decreasing the expression of Bcl-2-associated X protein (Bax) and Caspase-3 in rats [126]. Overall, the active components in EVs can be altered and their function optimized by pretreatment of MSCs.

### 4.4 Combining MSC-EVs with other technologies

Up to date, the medical applications of MSC-EVs were enhanced through interdisciplinary approaches that combined them with other technologies (Fig. 2). 3D graphene scaffolds and 2D graphene films have been used as substrates to culture hUCMSCs, improving their potential for industrial manufacture. The findings indicated notable variances in miRNA and various protein-protein (NEP, HSP70, etc.) expression levels between 3D-EVs and 2D-EVs. This modulation mitigated Aβ deposition by controlling the activities of α and β secretases [127]. Borneol is a type of compound that can be isolated from resins and has been traditionally utilized in Chinese medicine to enhance drug penetration through the blood-brain barrier (BBB). Ziconotide (ZIC) relieves severe chronic pain by blocking N-type voltage-sensitive calcium channels in neurons. Song *et al*. employed chemical modification to fuse BOR-modified liposomes with EVs and wrapped the ZIC in modified EVs (ZIC@EVs/LIP-BOR). They suggested that ZIC@EVs/LIP-BOR facilitates the drug delivery through the BBB and provides long-lasting relief for chronic pain in different peripheral nerve pain models [128]. These novel experiments can provide new ideas and rationales for enhancing the clinical application of MSC-EVs (Fig. 2) [127].

## 5. Challenges in MSC-EVs-based therapy for AD

EVs are widely used in preclinical studies owing to their multifaceted advantages and multiple modification methods; however, they still face great challenges in the process of product standardization and clinical promotion [129-132].

### 5.1 Heterogeneity in the production and extraction of EVs

At present, the development of EVs products primarily focuses on active substances such as DNA, miRNA and proteins. However, these active substances are affected by cell culture conditions and exhibit significant heterogeneity. To ensure product consistency, it is crucial to standardize the upstream culture process [133]. In addition, there is a lack of standardized methods for EVs extraction, and separating liposomes from EVs of similar sizes remains a challenge with most techniques. Common approaches such as ultracentrifugation (UC), inevitably compromises the integrity of the EVs, while ultrafiltration (UF) offers higher yield but lower purity [134]. Therefore, it is urgent to update and upgrade the extraction techniques of EVs. It has been shown that the topology of EV membrane proteins makes them targetable [135]. In addition, homogeneity of EVs can also be ensured by chemically synthesizing specific lipids that selectively adsorb particular EVs [136].

### 5.2 Optimizing the route of administration and dose of EVs

In preclinical studies, the common methods for administering EVs are intranasal administration and tail vein injection. Intranasal administration is a noninvasive approach widely utilized to effectively cross the BBB and rapidly deliver EVs to the brain [137]. Markoutsa *et al*. discovered that 1,1'-dioctadecyl-3,3,3',3'-tetramethy-lindotricarbocyanine iodide (DiR), can label EVs by binding to their lipophilic biomolecules, enabling tracking of their distribution in the body using infrared light emission [138]. *In vivo* imaging results showed that intranasally administered EVs can reach and accumulate in the brain and lungs within an hour [39]. On the other hand, EVs injected via the tail vein would take approximately one day to reach the brain, with the fluorescent signal peaking on the third day [139]. Following intravenous administration, a significant proportion of EVs tends to accumulate in the spleen and liver [140]. Additionally, determining the optimal EVs dosage is challenging as it depends on various factors, including the source of EVs, mode of administration, type of disease, and individual differences. We have summarized recent studies detailing the dosage and administration timing of EVs for different conditions (Table 1).

### 5.3 Current status and prospects of clinical research on MSC-EVs

Despite significant progress in preclinical studies of EVs, there have been few clinical applications to date. After reviewing the clinical database (www.clinicaltrials.gov), we found a clinical trial of MSC-EVs for mild to moderate AD patients was conducted at Shanghai Ruijin Hospital. The patients received low, medium, and high doses (5, 10, 20 μg) of MSC-EVs by nasal drip twice a week for 12 weeks, and their cognitive, motor, and neuroimaging abilities were evaluated periodically (NCT04388982). Although the results of this study are yet to be published, other clinical trials have shown promising results. In a clinical study conducted in Wuhan, China, seven COVID-19 patients were administered materialized MSC-EVs by nasal inhalation at a dose of 2×10^8^ particles per day for five consecutive days (NCT04276987). There were no adverse reactions or clinical instability during or after treatment, indicating the safety and stability of MSC-EVs as a potential clinical therapy. The stability and short-term safety of MSC-EVs gives us hope for clinical application [141]. Additionally, MSC-EVs are being studied in clinical trials for a range of diseases, including acute ischemic stroke (NCT05871463), liver cirrhosis (NCT03384433), and dry eye (NCT04213248), demonstrating the significant potential of cell-free therapy.

### 5.4 The quality control requirements and standardization of EVs product

The development of biological drugs in the early stages follows the principle of “the process is the product”. This means that standardized production processes result in more consistent EVs. For large-scale production lines, adherence to Good Manufacturing Practice (GMP) is crucial to maintaining the quality throughout the production process. Typically, the number of EVs can be quantified using Nanoparticle Tracking Analysis (NTA), while the purity of EVs is reflected in the ratio of total particles to total protein amount [142]. Early clinical trials in Phase 1 and Phase 2 focused on the safety of EVs products. It is only after significant results are demonstrated in the pivotal Phase 3 trials, EVs can be considered for approval as new drugs [143]. Once a drug enters the body, it may persist at a certain site, possibly impacting long-term safety. Therefore, it is essential to characterize the persistence and *in vivo* distribution of cell-based medicinal products (CBMP) in humans. Therefore, it is essential to characterize the persistence and *in vivo* distribution of cell-based medicinal products (CBMP) in humans. If there is sufficient evidence to demonstrate a product's potential to address an unsolved clinical problem, it can be designated as RMAT-qualified, resulting in accelerated approval and shortened research and development time.

## 6. Conclusion

Overall, MSC-EVs hold significant promise as cell-free therapies. Researchers have made advancements in enhancing the targeting and therapeutic effects of EVs through various modifications. However, ongoing studies need to address issues such as heterogeneity, optimal mode of administration, and dosage to maximize therapeutic efficacy. The development and clinical application of EV products still face several challenges. Ensuring quality control and standardization of EVs products is of paramount importance. Overcoming these challenges will serve as a crucial driving force for the clinical translation of MSC-EVs.
